# Preliminary study of AI-assisted diagnosis using FDG-PET/CT for axillary lymph node metastasis in patients with breast cancer

**DOI:** 10.1186/s13550-021-00751-4

**Published:** 2021-01-25

**Authors:** Zongyao Li, Kazuhiro Kitajima, Kenji Hirata, Ren Togo, Junki Takenaka, Yasuo Miyoshi, Kohsuke Kudo, Takahiro Ogawa, Miki Haseyama

**Affiliations:** 1 Graduate School of Information Science and Technology, Hokkaido University, N-14, W-9, Kita-ku, Sapporo, 060-0814 Japan; 2 Department of Radiology, Division of Nuclear Medicine and PET Center, Hyogo College of Medicine, 1-1 Mukogawa-cho, Nishinomiya, Hyogo 663-8501 Japan; 3 Department of Diagnostic Imaging, Graduate School of Medicine, Hokkaido University, Kita 15, Nishi 7, Kita-Ku, Sapporo, Hokkaido 060-8638 Japan; 4 Education and Research Center for Mathematical and Data Science, Hokkaido University, N-12, W-7, Kita-ku, Sapporo, 060-0812 Japan; 5 Department of Breast and Endocrine Surgery, Hyogo College of Medicine, 1-1 Mukogawa-cho, Nishinomiya, Hyogo 663-8501 Japan; 6 Global Center for Biomedical Science and Engineering, Faculty of Medicine, Hokkaido University, N-14, W-9, Kita-ku, Sapporo, 060-0814 Japan; 7 Faculty of Information Science and Technology, Hokkaido University, N-14, W-9, Kita-ku, Sapporo, 060-0814 Japan

**Keywords:** Breast cancer, Axillary lymph node, 2-[^18^f]FDG-PET/CT, AI-assisted diagnosis, Deep convolutional neural network

## Abstract

**Background:**

To improve the diagnostic accuracy of axillary lymph node (LN) metastasis in breast cancer patients using 2-[^18^F]FDG-PET/CT, we constructed an artificial intelligence (AI)-assisted diagnosis system that uses deep-learning technologies.

**Materials and methods:**

Two clinicians and the new AI system retrospectively analyzed and diagnosed 414 axillae of 407 patients with biopsy-proven breast cancer who had undergone 2-[^18^F]FDG-PET/CT before a mastectomy or breast-conserving surgery with a sentinel lymph node (LN) biopsy and/or axillary LN dissection. We designed and trained a deep 3D convolutional neural network (CNN) as the AI model. The diagnoses from the clinicians were blended with the diagnoses from the AI model to improve the diagnostic accuracy.

**Results:**

Although the AI model did not outperform the clinicians, the diagnostic accuracies of the clinicians were considerably improved by collaborating with the AI model: the two clinicians' sensitivities of 59.8% and 57.4% increased to 68.6% and 64.2%, respectively, whereas the clinicians' specificities of 99.0% and 99.5% remained unchanged.

**Conclusions:**

It is expected that AI using deep-learning technologies will be useful in diagnosing axillary LN metastasis using 2-[^18^F]FDG-PET/CT. Even if the diagnostic performance of AI is not better than that of clinicians, taking AI diagnoses into consideration may positively impact the overall diagnostic accuracy.

## Background

Breast cancer has been reported as the most prevalent cancer among women in western countries, and it causes the second greatest number of cancer-related deaths among females [1]. The treatments and prognoses of breast cancer depend on several factors including the size and grade of the tumor, the patient's endocrine (hormonal) receptor (ER) status and human epidermal growth factor receptor 2 (HER2) status, axillary lymph node (LN) involvement, and metastatic spread. Among these factors, the extent of axillary LN metastasis is regarded as the most reliable predictor of survival in breast cancer [2]. A determination of the patient's axillary nodal status before treatment can contribute to management decisions and is thus significant.

The 'gold standard' for diagnosing axillary LN involvement is a pathological examination of aspiration cytology, a sentinel LN biopsy (SLNB), and an axillary LN dissection (ALND); however, these are invasive methods. In contrast, the utility of noninvasive 2-deoxy-2-[^18^F]fluoro-D-glucose positron emission tomography/computed tomography (2-[^18^F]FDG-PET/CT) for the diagnosis of axillary LN metastasis in patients with breast cancer has been described by several research groups [3–9], one of which achieved a relatively low pooled sensitivity value of 60% and a quite high pooled specificity value of 97% [8].

To improve the accuracy of diagnoses of axillary LN metastasis by clinicians using 2-[^18^F]FDG-PET/CT, recent artificial intelligence (AI) technologies are worthy of consideration. Deep-learning technologies, which typically use deep convolutional neural networks (DCNNs), have been widely applied to the field of medical image analysis [10], including 2-[^18^F]FDG-PET/CT [11]. Although AI models trained with mass data can be competitive with experienced clinicians in some applications, in most cases, AI cannot outperform clinicians. This is due in part to the lack of well-annotated data. However, suboptimal AI models trained with a limited amount of data may not necessarily be useless.

In this study, we examined the practicability of using deep-learning technologies to improve the diagnosis of axillary LN metastasis with 2-[^18^F]FDG-PET/CT for breast cancer patients. We constructed an AI-assisted diagnosis system by developing a DCNN-based diagnosis method and a collaboration method blending AI and clinicians' diagnoses. The experimental results confirmed the effectiveness of the proposed AI-assisted diagnosis using deep-learning technologies.

## Materials and methods

### Patients

The appropriate review board at each institution (The Ethics Review Board of Hyogo College of Medicine, and The Ethics Committee at Hokkaido University Faculty of Medicine) approved this retrospective study, and the requirement for patient-informed consent was waived. We collected the data of 410 female patients with newly diagnosed invasive breast cancer who underwent pretreatment whole-body 2-[^18^F]FDG-PET/CT examinations before their surgery between September 2008 and September 2019. We excluded three patients with other existing diseases (malignant lymphoma, leukemia, and sarcoidosis). Seven patients had bilateral breast cancer, and thus a final total of 414 index breast cancers in 407 patients (28–90 years; mean ± SD 59.2 ± 14.0 years) were included in the study. The patient and tumor characteristics are summarized in Table 1. One hundred twenty-five patients (30.7%) underwent neoadjuvant chemotherapy (NAC) and/or hormonal therapy before the surgery. For the NAC, anthracycline-containing regimens, anthracycline followed by taxanes, or taxane-based regimens were administered. Hormonal therapy was given to the patients with hormone receptor-positive breast cancer, and the patients with HER2-positive breast cancer were treated with a trastuzumab-based regimen.

**Table 1:** Patient and tumor characteristics

	*n*	%
Total patients	407	
Age mean (range)	59.2 (28–90)	
Tumor location, right/left/bilateral	228/172/7	56.0%/42.3%/1.7%
NAC, yes/no	125/282	30.7%/69.3%
Total breast cancers	414	
Type of surgery		
Breast-conserving surgery	164	39.6%
Modified radical mastectomy	250	61.4%
Histology		
IDC	373	90.1%
Others		
(Myxoid/ILC/apocrine/metaplastic)	15/14/11/1	0.9%
Molecular phenotype		
Luminal A (ER + /HER2 − , Ki67 < 20%)	148	35.7%
Luminal B (ER + /HER2 − , Ki67 ≥ 20%)	120	29.0%
Luminal-HER2 (ER + /HER2 +)	43	10.4%
HER2-positive (non-luminal)	43	10.4%
Triple-negative	60	14.5%
Axillary lymph node metastasis		
Present	204	49.3%
Absent	210	50.7%
Diagnostic tool of axillary node		
SLNB	197	47.6%
ALND	12	2.9%
SLNB and ALND	59	14.3%
Aspiration cytology and ALND	60	14.5%
Aspiration cytology and SLNB	19	4.6%
Aspiration cytology, SLNB, and ALND	67	16.2%
TNM Stage (I/II/III)	140/217/57	33.8%/52.4%/13.8%

ALND: axillary lymph node dissection, ER: endocrine receptor, HER: human epidermal growth factor receptor, IDC: invasive ductal cancer, ILC: invasive lobular cancer, NAC: neoadjuvant chemotherapy, SLNB: sentinel lymph node biopsy, TNM: tumor node metastasis

The subtypes of the 414 tumors were luminal A (ER + /HER2 − , Ki67 < 20%) in 148 tumors (14.3%), luminal B (ER + /HER2 − , Ki67 ≥ 20%) in 120 (35.7%) tumors, luminal-HER2 (ER + /HER2 +) in 43 (10.4%) tumors, HER2-positive (non-luminal) in 43 (10.4%) tumors, and triple-negative in 60 (14.5%) tumors. Regarding the tumor node metastasis (TNM) stage, the tumors of 140 patients (33.8%) were stage I, those of 217 (52.4%) were stage II, and those of the other 57 (13.8%) were stage III.

Among the 414 axillae, 204 (49.3%) were diagnosed pathologically as having axillary LN metastasis. The axillary node metastasis was confirmed by the overall assessment of aspiration cytology, SLNB, and ALND. Histopathologic characteristics were determined based on the samples obtained by core needle biopsy and surgical resection findings. All the 125 patients who underwent NAC proved to have LN metastasis by the aspiration.

### 2-[^18^F]FDG-PET/CT

All 2-[^18^F]FDG-PET/CT examinations were performed by using one of four PET/CT scanners: a Gemini GXL (Philips Medical Systems, Eindhoven, The Netherlands) (n = 283), Gemini TF (Philips Medical Systems) (n = 72), Ingenuity TF (Philips Medical Systems) (n = 26), and Discovery IQ5 (GE Healthcare, Waukesha, WI, USA) (n = 26). The clinical parameters are shown in Table 2.

**Table 2:** Clinical parameters of PET/CT scanners

Scanner	Gemini GXL	Gemini TF64	IQ5	Ingenuity TF
Vendor	Philips	Philips	GE	Philips
*CT scanning*				
Tube voltage	120 kV	120 kV	120 kV	120 kV
Effective tube	current auto-mA up to 120 mA	100 mA	12 ~ 390 mA (Smart mA: Noise Index 25)	100 mA (variable by Dose Right)
Detector configuration	16 × 1.5 mm	64 × 0.625 mm	16 × 1.25 mm	64 × 0.625 mm
Slice thickness, mm	2	2	3.75	2
Transverse FOV, mm	600	600	700	600
*PET scanning*				
2-[^18^F]FDG injection dose, MBq/kg	4	3	3.7	3.7
Scan time for each bed, mm	90	90	180	90
TOF	No	Yes	No	Yes
*PET reconstruction*				
Reconstruction	LOR-RAMLA	3D-OSEM	3D-OSEM + PSF + Q-clear	3D-OSEM
Iterations	2	3	4	3
Subsets	n/a	33	12	33
Smoothing	n/a	n/a	Gaussian	n/a
FWHM of filter, mm	5.3	4.7	4.3	4.8
Matrix	144 × 144	144 × 144	192 × 192	144 × 144
Pixel size, mm	4 × 4 × 4	4 × 4 × 4	3.125 × 3.125 × 3.125	4 × 4 × 4

2-[^18^F]FDG: 2-deoxy-2-[^18^F]fluoro-D-glucose, FWHM: full-width at half maximum, LOR-RAMLA: line-of-response row-action maximum likelihood algorithm, OSEM: ordered-subset expectation maximization, PSF: point spread function, TOF: time of flight

### Human diagnosis

All 2-[^18^F]FDG-PET/CT images were retrospectively reviewed by one experienced reader (12 years of experience with oncologic 2-[^18^F]FDG-PET/CT; referred to as clinician A hereinafter) and one reader (2 years of experience with oncologic 2-[^18^F]FDG-PET/CT; referred to as clinician B hereinafter), both of whom had no knowledge of the other imaging results or clinical and histopathologic data other than the presence of breast cancer. Because several groups have reported that the diagnostic performances of qualitative and quantitative assessments were not significantly different [9, 12, 13], we used a qualitative assessment in this study. The diagnostic certainty of assessing axillary LN metastasis was visually graded as 1 (definitely absent), 2 (probably absent), 3 (indeterminate), 4 (probably present), and 5 (definitely present). An LN was graded as 4 or 5 if it showed 2-[^18^F]FDG uptake greater than that of the reference background. A non-elevated PET signal or one considered compatible with physiological lymphatic uptake was rated as grade 1 or 2.

### AI diagnosis

DCNNs, which have been the most popular AI model in recent years, have enabled tremendous achievements in various medical image analysis tasks [14]. However, the task in the present study is quite different from the previous tasks handled with DCNNs. In general diagnosis tasks of medical images, DCNNs are usually trained to distinguish abnormality from normality by recognizing one specific type of lesion. In our present investigation, the objects of interest are patients diagnosed as having breast cancer, which requires the DCNN model to distinguish between breast cancer and axillary LN metastasis. DCNN models are faced with a dilemma in such a task since breast cancer and axillary LN metastasis have similar characteristics in terms of 2-[^18^F]FDG uptake on PET images. In addition, in CT images, the anatomical structures of breast cancer and axillary LN metastasis are ambiguous to DCNN models without a human's technical knowledge. It is thus a challenging task for DCNN models to diagnose axillary LN metastasis with PET/CT images.

To overcome this problem, we designed a deep 3D residual convolutional neural network (CNN) equipped with an attention mechanism. The residual network is one of the most significant CNN structures and has been considered to be generally effective [15]. A 3D CNN can analyze PET/CT images without a deficiency of spatial information, which occurs with a general 2D CNN. The attention mechanism also enables the network to pay closer attention to regions that are truly meaningful to diagnoses, i.e., the locations at which the breast cancer and axillary LN metastases appear [16].

We constructed the network to perform a three-class classification: (1) no breast cancer, (2) breast cancer but no axillary LN metastasis, and (3) axillary LN metastasis of breast cancer. The network receives only the chest regions of the PET/CT images as inputs rather than the whole-body PET/CT images. The PET image and the CT image are concatenated as different channels to be fed into the network. One side of each PET/CT image (left chest or right chest; separated by the central line) is regarded as one training sample, which eliminates the need for healthy control subjects, since a side with no breast cancer can be used as a healthy side. In this manner, a total of 814 samples were obtained from the 407 patients with breast cancer: 400 normal samples, 210 breast cancer samples with no axillary LN metastasis, and 204 axillary LN metastasis samples. The three-class classification network was trained with the 814 samples.

**Fig. 1:**
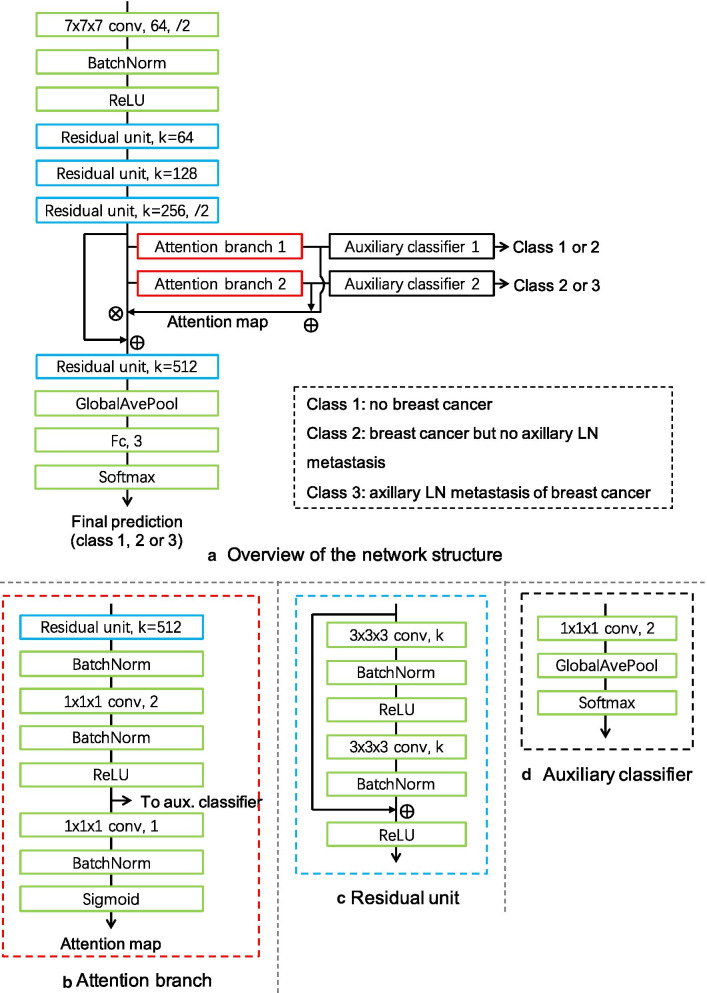
The network structure of the 3D CNN. (**a**) Overview of the network structure. (**b**) Structure of attention branch. (**c**) Structure of residual unit. (**d**) Structure of auxiliary classifier

The network structure of the 3D CNN is shown in Fig. 1. The network mainly contains four residual units and two attention branches. Each attention branch is followed by an auxiliary classifier. The two auxiliary classifiers are trained for different tasks. One is trained to differentiate between class (1) and class (2) so that the corresponding attention branch can produce an attention map that focuses on the region related to the breast cancer. The other one is for differentiating between class (2) and class (3), and the attention branch produces an attention map that focuses on the region related to the axillary LN metastasis. The two attention maps are summed to guide the final three-class classification. The network was trained by a stochastic gradient descent (SGD) optimizer with a learning rate of 0.001. The cross-entropy losses of the main task and two auxiliary tasks were weighted equally.

Before the network was trained, the samples were normalized for more accurate and faster processing by the network. The PET images were clipped by using a maximum standardized uptake value (SUVmax) cutoff of 6, i.e., voxels with an SUV value > 6 were assigned 6, and then normalized to [0, 1]. Similarly, the CT images were clipped by a low Hounsfield unit (HU) cutoff of − 100 and a high HU cutoff of 200 and then normalized to [0, 1]. The cutoff values for the PET images and the CT images were determined by joint empirical and experimental estimations.

### The AI-assisted diagnosis

To use the AI model as an assistant, we blended the diagnoses from the AI model with the clinicians' diagnoses. Since the AI model is not as reliable as the clinicians due to the limited amount of training data, the blending was biased toward the clinicians. Specifically, the graded clinicians' diagnoses were first converted into diagnostic probabilities of having axillary LN metastasis according to the diagnostic certainty: grade 1 corresponds to 0%, grade 2 to 25%, grade 3 to 50%, grade 4 to 75%, and grade 5 to 100%. The diagnostic probability from the clinicians (which we refer to as $P_{{{\text{cli}}}}$) was then blended with the diagnostic probability from the AI model (which we refer to as $P_{{{\text{ai}}}}$) using a confidence weight $\alpha = {\text{Max}} \left( {P_{{{\text{cli}}}} ,1 - P_{{{\text{cli}}}} } \right)$ as the following equation:

$$P_{{{\text{blend}}}} = \alpha \times P_{{{\text{cli}}}} + \left( {1 - \alpha } \right) \times P_{{{\text{ai}}}} .$$

Finally, the blend diagnostic probability was converted back into the graded diagnosis in the following manner: probabilities of 0–20% are regarded as grade 1, 21–40% as grade 2, 41–60% as grade 3, 61–80% as grade 4, and 81–100% as grade 5.

**Fig. 2:**
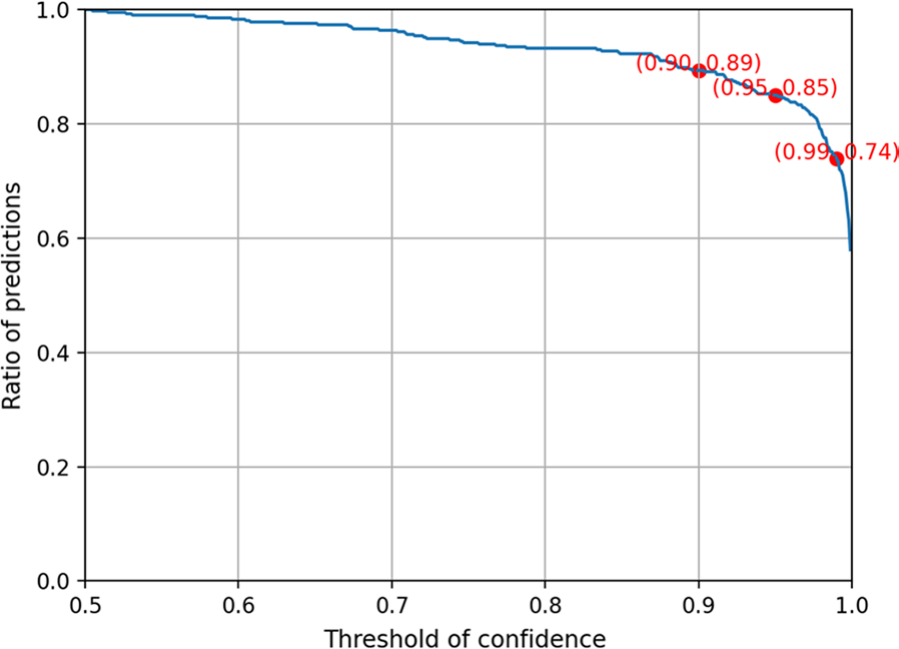
The relationship between the threshold and the ratio of predictions with a confidence value larger than the threshold on the 414 samples with breast cancers

Based on a generally valid assumption in the field of deep learning that predictions with high confidence made by DCNN models tend to be more accurate than those with low confidence, we did not adopt diagnoses with relatively low confidence from the AI model for the AI-assisted diagnosis in this study. Here, 'confidence' denotes ${\text{Max}} \left( {P_{{{\text{ai}}}} ,1 - P_{{{\text{ai}}}} } \right)$. To determine an appropriate confidence threshold, we studied the relationship between the threshold and the ratio of predictions with a confidence value larger than the threshold on the 414 samples with breast cancers. From Fig. 2 illustrating the relationship, it can be seen that the ratio decreases slowly until the threshold increases to around 0.95, and then the ratio decreases much faster. We therefore chose 0.95 as the confidence threshold in this study.

In the AI-assisted diagnosis system, we can quantify how the AI assistance impacts a clinician's diagnoses as follows. For diagnoses of grade 1 and grade 5, the AI assistance has no effect since the confidence weight ${\upalpha }$ is 1. For diagnoses of grade 2 and grade 4, the AI model can either agree with the clinician and enhance the diagnostic certainty, i.e., modify the grade to 1 or 5, or query the clinician's diagnosis and modify the grade to 3. For diagnoses of grade 3, the AI model can help the clinician to make to some extent definite diagnoses and modify the grade to 2 or 4. Note that these cases are limited to samples selected by the confidence threshold. The AI diagnoses screened out by the threshold are not taken into consideration, and thus the clinician's diagnoses are considered the final diagnoses for these samples.

### Statistical analyses

A fivefold cross-validation was conducted on the 407 patients. For the AI model, a receiver operating characteristic (ROC) curve and an area under curve (AUC) value of the ROC curve were calculated for evaluation since the diagnoses from the AI model are continuous probabilities. However, the diagnoses from the two clinicians are of five grades so that it is less meaningful to compare the ROC curves between the clinicians and the AI model. To compare the performances of the AI model and the clinicians and evaluate the performance of the AI-assisted diagnosis, we used sensitivity, specificity, and accuracy as evaluation metrics.

## Results

The evaluations were performed mainly on the 414 samples of the half-chests with breast cancers. The mean size of 414 axillae was 5.5 ± 5.0 mm (range 0–35 mm, median 4 mm). The performances of the human (clinicians') diagnoses, AI diagnoses, and AI-assisted diagnoses are presented as follows.

### Human diagnosis

In general, LNs graded as 4 and 5 are considered positive, and on the 414 samples, the side-based sensitivity, specificity, and accuracy values of clinician A's reading for diagnosing axillary LN metastasis were 59.8% (122/204), 99.0% (208/210), and 79.7% (330/414), respectively. When including LNs of grade 3 as positive, the side-based sensitivity, specificity, and accuracy of clinician A's reading were 74.0% (151/204), 96.7% (203/210), and 85.5% (354/414), respectively.

For clinician B, on the 414 samples, the side-based sensitivity, specificity, and accuracy when grades 4 and 5 were considered positive were slightly lower than the results of clinician A, at 57.4% (117/204), 99.5% (209/210), and 78.7% (326/414), respectively. The side-based sensitivity, specificity, and accuracy when grades 3, 4, and 5 were considered positive were 68.6% (140/204), 99.0% (208/210), and 84.1% (348/414), respectively.

### AI diagnosis

**Fig. 3:**
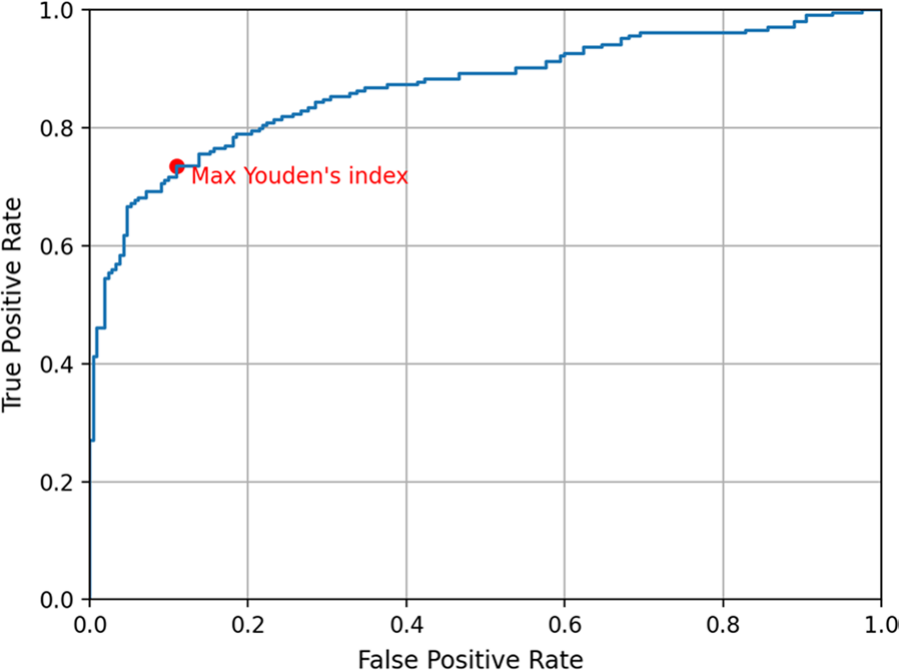
The side-based ROC curve of the AI diagnosis for axillary LN metastasis on the 414 samples

For the 414 samples, the side-based AUC of the AI diagnosis for axillary LN metastasis was 0.868. The ROC curve is shown in Fig. 3. The maximum Youden's index (*J* = sensitivity + specificity − 1) is marked on the curve. The side-based sensitivity, specificity, and accuracy values at the maximum Youden's index were 73.5% (150/204), 89.0% (187/204), and 81.4% (337/414), respectively.

### AI-assisted diagnosis

Table 3 compares the performances of the human diagnoses and AI-assisted diagnoses for axillary LN metastasis on the 414 samples. The AI-assisted diagnosis results were obtained by the aforementioned blending method in which the diagnoses of grades 2, 3, and 4 from the clinicians may be modified by the AI model. The side-based values of sensitivity, specificity, and accuracy of the two clinicians with and without AI assistance under different positive standards are listed in Table 3 to demonstrate the effect of AI assistance.

**Table 3:** The side-based sensitivity, specificity, and accuracy values of the human (clinicians') diagnoses and AI-assisted diagnoses on the 414 samples

Graded as positive	Clinicians with/without AI assistance	Sensitivity (%)	Specificity (%)	Accuracy (%)
3, 4, 5	Clinician A w/o AI	74.0	96.7	85.5
	Clinician A w/ AI	76.5	94.3	85.5
	Clinician B w/o AI	68.6	99.0	84.1
	Clinician B w/ AI	68.6	99.0	84.1
4, 5	Clinician A w/o AI	59.8	99.0	79.7
	Clinician A w/ AI	68.6	99.0	84.1
	Clinician B w/o AI	57.4	99.5	78.7
	Clinician B w/ AI	64.2	99.5	82.1
5	Clinician A w/o AI	37.3	100	69.1
	Clinician A w/ AI	54.9	99.5	77.5
	Clinician B w/o AI	33.8	100	67.4
	Clinician B w/ AI	54.4	100	77.5

As shown in Table 3, when considering grades 4 and 5 as positive, the AI assistance brought significant improvements in sensitivity and accuracy while keeping the extremely high specificity value unchanged. The two clinicians' sensitivities were increased by 8.8% and 6.8%, and the accuracies were increased by 4.4% and 3.4%, respectively. These improvements indicate that the AI assistance helped the clinicians make relatively accurate diagnoses for the ambiguous samples graded as 3 by the clinicians. When considering only grade 5 as positive, the diagnoses of the clinicians were also improved considerably by the AI assistance in sensitivity (increased by 17.6% and 20.6%, respectively) and accuracy (increased by 8.4% and 10.1%, respectively). The improvements were gained by enhancing the diagnostic certainty with the AI assistance. However, when considering grades 3, 4, and 5 as positive, the AI assistance hardly affected the clinicians' performances. This result implies that the AI model cannot accurately diagnose the positive samples graded as 2 by the clinicians and cannot recognize more negative samples than the clinicians. As a whole, according to Table 3, the effects of the AI assistance on the two clinicians were substantially consistent.

Tables 4 and 5 elaborate the effect of AI assistance on the diagnoses made by the two clinicians, i.e., which grades the samples were considered by the clinicians and reconsidered with AI assistance. Samples graded as 1 and 5 by the clinicians were not included in the tables since grades of these samples were unaffected. In the tables, a number marked by the asterisk '*' denotes diagnoses corrected by the AI assistance including (1) false-positive and false-negative samples reconsidered as grade 3, and (2) samples of grade 3 reconsidered correctly as grade 2 or grade 4. In contrast, a number marked '**' denotes mistakenly reconsidered diagnoses including (1) true-positive and true-negative samples reconsidered as grade 3, and (2) grade 3 samples reconsidered mistakenly as grade 2 or grade 4. It is clear in Tables 4 and 5 that the major contribution of the AI assistance came from helping the clinicians diagnose the ambiguous grade 3 samples.

**Table 4:** Details of how the AI assistance affected the diagnoses made by clinician A

Regraded with AI assistance	Grade by clinician A					
	Grade 2 127		Grade 3 34		Grade 4 48	
	Positive 34	Negative 93	Positive 29	Negative 5	Positive 46	Negative 2
Grade 1	19	63				
Grade 2	7	22	3**	3*		
Grade 3	8*	8**	5	2	3**	
Grade 4			21*		7	1
Grade 5					36	1

‘*’ denotes diagnoses corrected by the AI assistance including (1) false-positive and false-negative samples reconsidered as grade 3, and (2) samples of grade 3 reconsidered correctly as grade 2 or grade 4

‘**’ denotes mistakenly reconsidered diagnoses including (1) true-positive and true-negative samples reconsidered as grade 3, and (2) grade 3 samples reconsidered mistakenly as grade 2 or grade 4

**Table 5:** Details of how the AI assistance affected the diagnoses made by clinician B

Regraded with AI assistance	Grade by clinician B					
	Grade 2 12		Grade 3 24		Grade 4 49	
	Positive 8	Negative 4	Positive 23	Negative 1	Positive 48	Negative 1
Grade 1	2	2				
Grade 2	3	2	3**			
Grade 3	3*		5		1**	1*
Grade 4			15*	1**	5	
Grade 5					42	

‘*’ denotes diagnoses corrected by the AI assistance including (1) false-positive and false-negative samples reconsidered as grade 3, and (2) samples of grade 3 reconsidered correctly as grade 2 or grade 4

‘**’ denotes mistakenly reconsidered diagnoses including (1) true-positive and true-negative samples reconsidered as grade 3, and (2) grade 3 samples reconsidered mistakenly as grade 2 or grade 4

Some supplementary results are provided for further analysis. Additional file 1: Fig. 1 shows the side-based ROC analysis of AI diagnosis on samples of the two scanner groups. Additional file 1: Fig. 2 shows the side-based ROC analysis of the AI diagnosis on two sides of the chest. Details are described in the Supplementary document.

## Discussion

2-[^18^F]FDG-PET/CT can be a noninvasive means for diagnosing LN metastasis. It imposes less burden on patients than invasive means such as SLNB and ALND. However, despite the very high specificities (99.0% and 99.5%) of 2-[^18^F]FDG-PET/CT observed in this study, the sensitivities of the human diagnosis with FDG-PET/CT for axillary LN metastasis were quite poor (59.8% and 57.4%). Similar results have been reported by other groups [3–9]. To improve the sensitivity, we constructed an AI-assisted diagnosis system. In the system, an AI model was trained to diagnose axillary LN metastasis with PET/CT images. The AI model underperformed the two clinicians, whereas with a collaboration method, the AI model helped the clinicians as an assistant to improve the diagnostic accuracy. Such assistance may be promising in clinical applications of AI [17].

Our present findings demonstrated that the proposed AI-assisted diagnosis system contributed mainly to diagnoses for ambiguous cases graded as 3 by the clinicians. As shown in Tables 4 and 5, 24/34 and 15/24 samples of grade 3 were diagnosed correctly with the AI assistance, whereas there were relatively small numbers of incorrect diagnoses at 3/34 and 4/24. For the grade 2 and grade 4 samples, the AI assistance could query the human diagnoses, but it failed to improve the diagnostic accuracy.

On the other hand, the AI assistance also helped the clinicians enhance the diagnostic certainty of their diagnoses of grades 2 and 4, which was confirmed by the results, but such assistance may not truly affect the clinical diagnostic accuracy. For the grade 1 and grade 5 samples, we did not use the AI diagnosis because we observed that doing so reduced the diagnostic accuracy. In short, our present results indicate that samples that the clinicians mistakenly diagnosed were also difficult for the AI model — especially the numerous false negatives.

Nevertheless, there were still some false-negative diagnoses that were made by the clinicians and queried by the AI model. Additional file 1: Fig. 3 shows a false-negative sample diagnosed by clinician A. The clinician gave grade 2, whereas the AI model gave a positive diagnosis. As a result, the diagnosis was modified to grade 3 by the AI-assisted diagnosis system. The patient was a 67-year-old woman with a Luminal B (HER2-negative)-type invasive ductal carcinoma (solid ductal cancer, ER 100%, PR 90%, HER2 1 + , Ki-67 20%, grade 1, T2N1M0, stage IIB) and ipsilateral axillary LN metastasis diagnosed by aspiration cytology. After neoadjuvant chemotherapy, she received breast-conserving surgery including an SLNB and ALND.

In light of the limited number of patients used to train the AI model in this study, a larger contribution of AI assistance may be promising if a greater number of patients are made available for training AI models. This is also implied by the results on the two scanner groups shown in Additional file 1: Fig. 1. The performance of the AI diagnosis was much better for the group examined with the Gemini GXL compared to the group examined by the Gemini TF, Ingenuity TF or Discovery IQ5. Because the 4 scanners had relatively similar spatial resolutions (Table 2) with FWHM ranging from 4.3 to 5.3 mm, the different performance of the AI diagnosis between scanners may be ascribed to the biased distribution of examination scanners. In cases of well-distributed examination scanners, we speculate that the performance of the AI diagnosis on the group of three scanners would not be worse than that for the Gemini GXL since the former scanners have better imaging quality than the Gemini GXL. Considering the limited spatial resolutions of PET, partial volume correction would have potential to improve sensitivity for small lesions, whereas it would also increase false-positive cases and thus degrade specificity. The sensitivity would be increased if the data were analyzed after the lymph nodes below the instrument resolution (e.g., < 10 mm) are excluded.

Due to limited performances and some other issues [18], AI cannot replace human clinicians completely in most clinical diagnoses. However, AI assistance can be useful in saving clinicians' time and/or improving diagnostic performance [19]. In the present study, the AI model which underperformed the clinicians showed an ability to diagnose cases that the clinicians considered indeterminate, with an AUC value of 0.903. This performance was even better than that on all of the samples, which indicates that the AI model has a different perspective from clinicians for diagnoses or can perceive some minute details. Such AI assistance may be desirable despite the difficulty in comprehensively interpreting how AI models make diagnoses.

Our study has several limitations, including its retrospective design, which may limit the generalization of the derived conclusions and may have caused statistical errors. Moreover, although a node-by-node-based analysis is ideal, it was difficult to correlate any given LN depicted by imaging with the same node in a dissection specimen. Therefore, the correlation between imaging results and pathological findings based on a side may be more reasonable for this type of study. Because the number of each subtype of breast cancer was small, we could not analyze the diagnostic performance of AI for each subtype, respectively. In addition, as mentioned above, it was difficult to interpret the inference process of the AI model, which may hinder the AI model from gaining more trust. Although some approaches have been proposed to locate the regions that have the greatest impacts on AI's decisions [20, 21], we observed herein that the localization can hardly be precise and thus gave poor hints. The best collaboration method between AI and clinicians merits further consideration and should be validated on a larger dataset.

## Conclusion

Although the AI model trained in this study cannot outperform clinicians, the proposed AI-assisted diagnosis system can improve the diagnostic accuracy of human diagnosis mainly by assisting in the diagnoses of indeterminate patients. However, for hard false negatives, the AI model provides poor assistance. Future studies with more sufficient and well-distributed data may be informative and further improve the diagnostic performance.

## Supplementary information

**Additional file 1**

## Data Availability

The corresponding author can be contacted for requests regarding the data and material.

## Abbreviations

ALND: Axillary lymph node dissection
AUC: Area under curve
DCNN: Deep convolutional neural network
ER: Endocrine receptor
2-[^18^F]FDG-PET/CT: 2-Deoxy-2-[^18^F]fluoro-D-glucose positron emission tomography/computed tomography
HER2: Human epidermal growth factor receptor 2
HU: Hounsfield unit
LN: Lymph node
NAC: Neoadjuvant chemotherapy
ROC: Receiver operating characteristic
SLNB: Sentinel lymph node biopsy
SUVmax: Maximum standard uptake value
TNM: Tumor node metastasis
