# Supplementary material for: Preliminary study of AI-assisted diagnosis using FDG-PET/CT for axillary lymph node metastasis in patients with breast cancer
Source: EJNMMI Res. 2021 Jan 25;11:10. doi: 10.1186/s13550-021-00751-4 (PMC7835273; doi:10.1186/s13550-021-00751-4)
Supplement: Supplementary file 1 — Additional file 1 [file 13550_2021_751_MOESM1_ESM.docx]

***Supplementary results***

For a further evaluation the AI diagnoses and the AI-assisted diagnoses, some supplementary results are provided as follows. First, we observed an effect of the various PET/CT scanners on the diagnostic accuracy of the AI model. We divided the four PET/CT scanners used in this study into two groups based on the imaging quality that they provide. The Gemini GXL scanner (which has imaging quality inferior to the other scanners) comprised one group, and the other three scanners comprised the other group. The side-based ROC curves of AI diagnosis for the two groups are shown in Supplementary Figure 1. The AUC values of the two ROC curves were 0.887 for the Gemini GXL and 0.826 for the other scanners. Our unexpected finding that the diagnoses obtained with the inferior scanner were more accurate may be explained by the biased data. Since 283 examinations of the total 407 2-[^18^F]FDG-PET/CT examinations were performed using the Gemini GXL scanner, the training of the AI model was biased toward the samples of the dominant scanner so that it underperformed on the other samples.

The accuracy of the AI diagnosis may also be influenced by the primary tumor characteristics of the breast cancer. We observed an effect of the SUVmax of the primary tumor on the diagnostic accuracy of the AI model. We divided the samples into three groups according to the primary tumor SUVmax: group (1) of 150 samples with 0 $\leq$ SUVmax $<$ 3, group (2) of 142 samples with 3 $\leq$ SUVmax $<$ 6, and group (3) of 122 samples with 6 $\leq$ SUVmax. The side-based AUCs of group (1), group (2) and group (3) were 0.691, 0.874 and 0.906, respectively. Obviously, the AI model performed better on samples with higher primary tumor SUVmax. The results can be explained by the three-class training process that involved also normal samples. Since samples of axillary LN metastasis must have breast cancer, the existence of breast cancer may be interpreted by the AI model as an essential characteristic or precondition for axillary LN metastasis. Higher SUVmax can facilitate the recognition of breast cancer, and thus axillary LN metastasis of those samples are easier to recognize for the AI model.

Considering the different environments of the two sides of the chest, especially in PET images, we also evaluated the AI diagnosis on each side. Supplementary Figure 2 shows the side-based ROC curves of which the AUC values were 0.891 (left side) and 0.852 (right side). The results seemed again unexpected because the performance on the left side (in which the uptake values in the heart region may produce a disturbance) were expected to be not better than that on the right side. We do not have a plausible explanation for this result; moreover, the results of 414 samples were not statistically meaningful enough.

The effect of AI assistance on the diagnostic performance depended on the performance of AI diagnosis on samples graded as 2, 3, and 4 by the clinicians. The side-based AUCs of the AI diagnoses on samples correctly graded as 2 or 4 by clinician A and samples graded as 3 by clinician A were 0.923 and 0.903, respectively, which were clearly better than the side-based AUCs for all 414 samples. These results explained why the AI assistance improved the diagnostic performance.

Finally, we provide some results of 814 samples including both sides of the 407 patients in Supplementary Table. With the introduction of the 400 negative samples without breast cancer, the AI assistance showed a further contribution to specificity compared to the results obtained with 414 samples.

　　 A representative case is shown in Supplementary Figure 3.

Supplementary Figure 1


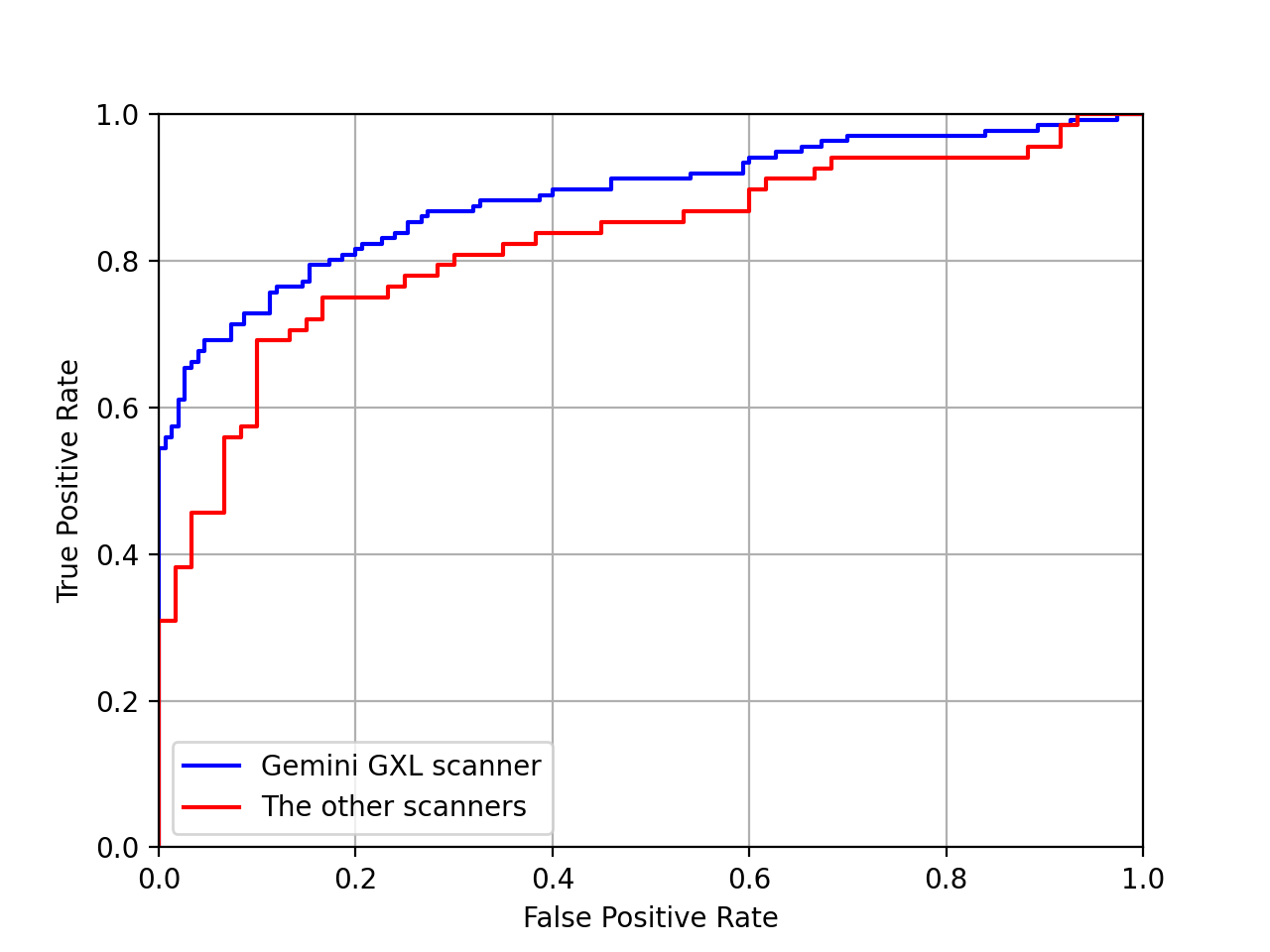


The side-based ROC curves of AI diagnosis on samples of the two scanner groups.

Supplementary Figure 2


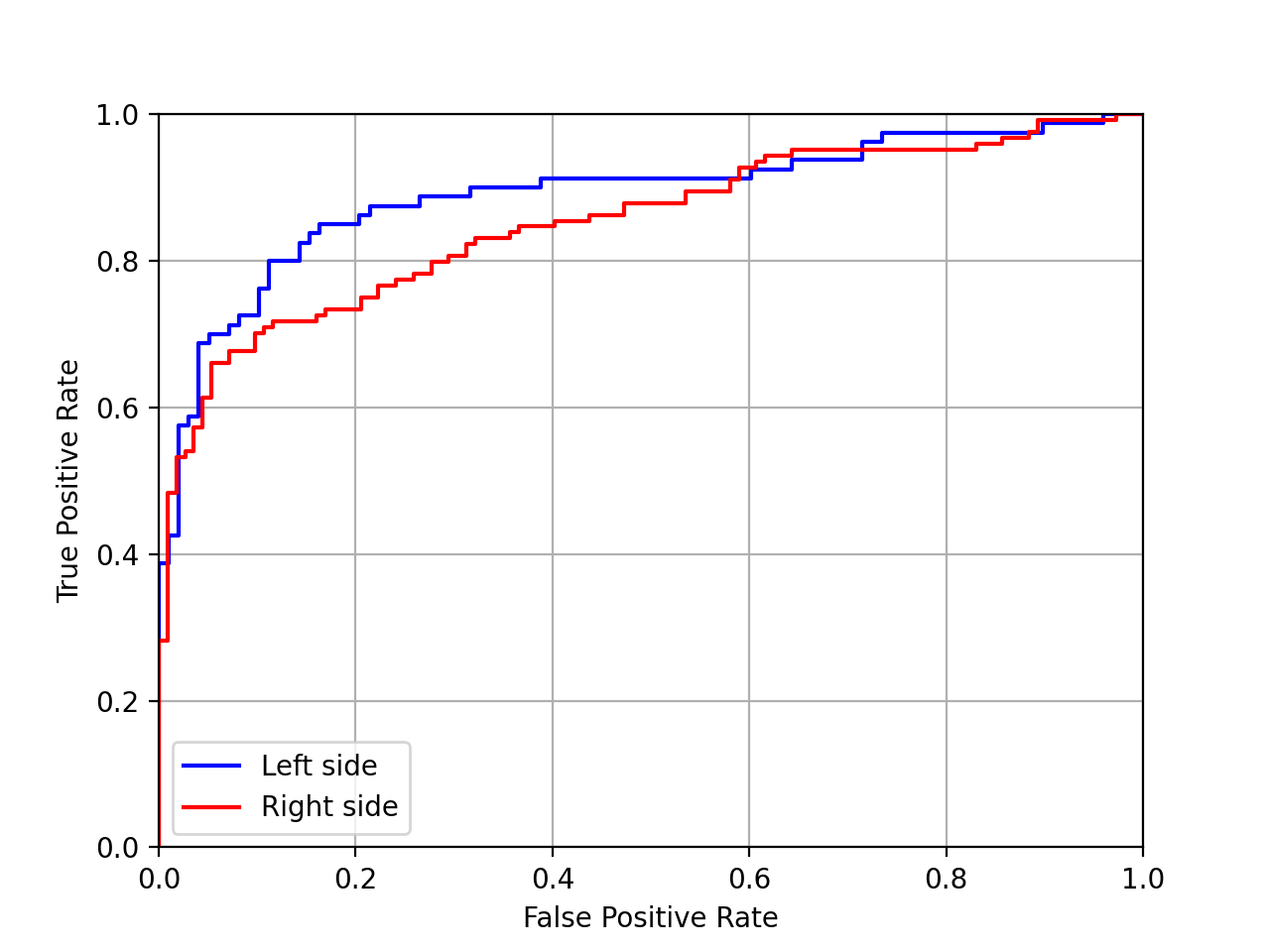


The side-based ROC curves of the AI diagnosis on two sides of the chest.

Supplementary Figure 3


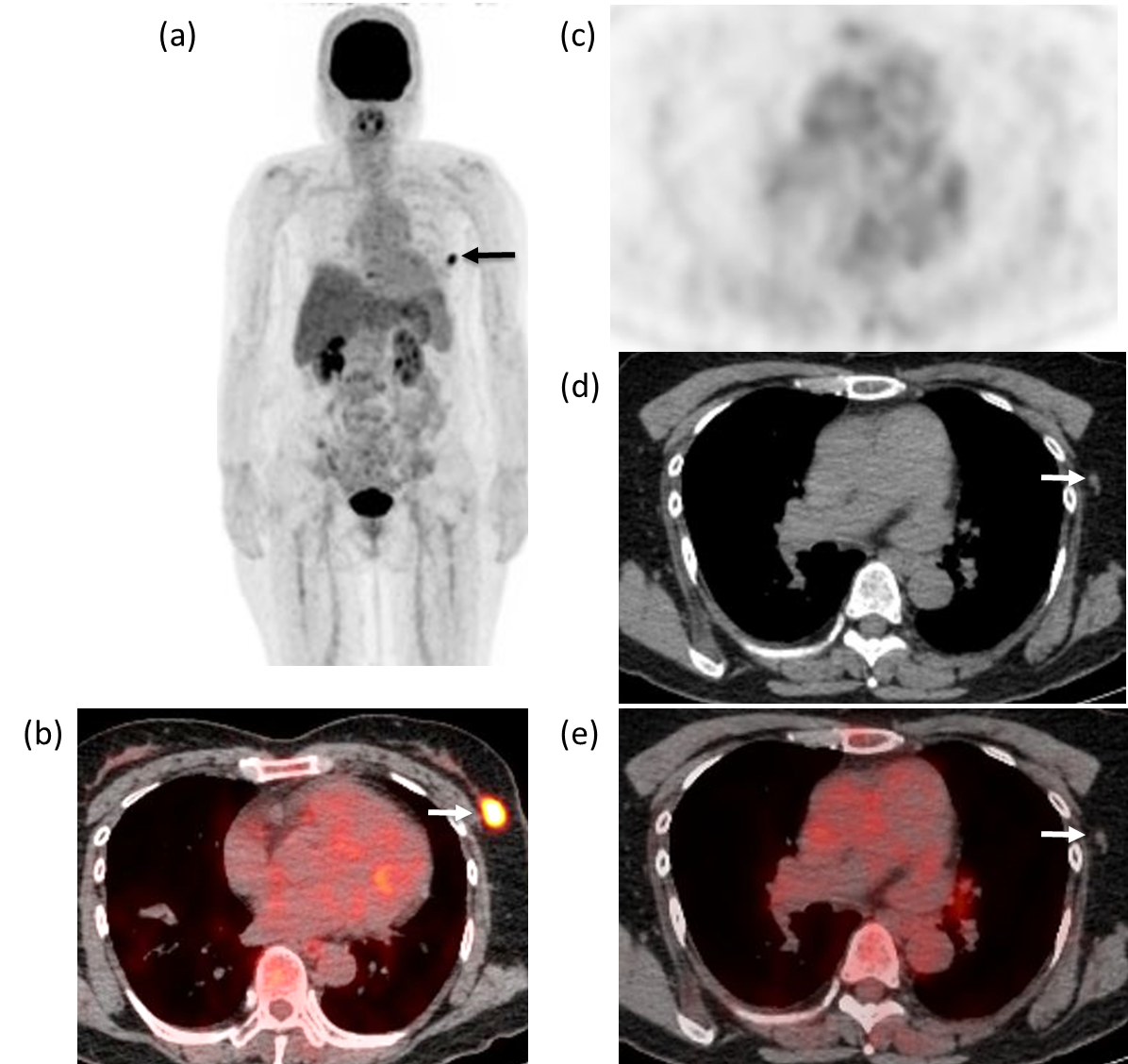


A positive sample that clinician A graded as 2 (probably negative) and the AI model diagnosed as positive. **(a)** Maximum intensity projection (MIP) from 2-[^18^F]FDG-PET. **(b)** Fused axial 2-[^18^F]FDG-PET/CT showing moderate 2-[^18^F]FDG uptake in the left breast tumor measuring 23 mm (*arrow*). **(c)** Axial 2-[^18^F]FDG-PET. **(d)** Axial CT. **(e)** Fused 2-[^18^F]FDG-PET/CT showing no abnormal 2-[^18^F]FDG uptake in a left tiny (4-mm) axillary LN (*arrow*).

**Supplementary Table**

The side-based sensitivities, specificities and accuracies of human diagnosis and AI-assisted diagnosis on the 814 samples

| **Graded as positive** | **Clinicians with/without AI assistance** | **Sensitivity** | **Specificity** | **Accuracy** |
| --- | --- | --- | --- | --- |
| 3, 4, 5 | Clinician A w/o AI | 74.0% | 96.9% | 91.2% |
|  | Clinician A w/ AI | 76.5% | 97.0% | 91.9% |
| 4, 5 | Clinician A w/o AI | 59.8% | 99.2% | 89.3% |
|  | Clinician A w/ AI | 68.6% | 99.3% | 91.6% |
